# Effects of Anterior Thalamic Nucleus Deep Brain Stimulation in Chronic Epileptic Rats

**DOI:** 10.1371/journal.pone.0097618

**Published:** 2014-06-03

**Authors:** Luciene Covolan, Antônio-Carlos G. de Almeida, Beatriz Amorim, Clarissa Cavarsan, Maisa Ferreira Miranda, Mayra C. Aarão, Ana Paula Madureira, Antônio M. Rodrigues, José N. Nobrega, Luiz E. Mello, Clement Hamani

**Affiliations:** 1 Disciplina de Neurofisiologia, Universidade Federal de São Paulo, São Paulo, Brazil; 2 Laboratório de Neurociência Experimental e Computacional, Universidade Federal de São João del-Rei, São João del-Rei, Brazil; 3 Behavioural Neurobiology Laboratory, Centre for Addiction and Mental Health, Toronto, Canada; 4 Division of Neurosurgery, Toronto Western Hospital, University of Toronto, Toronto, Canada; Emory University, Georgia Institute of Technology, United States of America

## Abstract

Deep brain stimulation (DBS) has been investigated for the treatment of epilepsy. In rodents, an increase in the latency for the development of seizures and status epilepticus (SE) has been reported in different animal models but the consequences of delivering stimulation to chronic epileptic animals have not been extensively addressed. We study the effects of anterior thalamic nucleus (AN) stimulation at different current intensities in rats rendered epileptic following pilocarpine (Pilo) administration. Four months after Pilo-induced SE, chronic epileptic rats were bilaterally implanted with AN electrodes or had sham-surgery. Stimulation was delivered for 6 h/day, 5 days/week at 130 Hz, 90 µsec. and either 100 µA or 500 µA. The frequency of spontaneous recurrent seizures in animals receiving stimulation was compared to that recorded in the preoperative period and in rats given sham treatment. To investigate the effects of DBS on hippocampal excitability, brain slices from animals receiving AN DBS or sham surgery were studied with electrophysiology. We found that rats treated with AN DBS at 100 µA had a 52% non-significant reduction in the frequency of seizures as compared to sham-treated controls and 61% less seizures than at baseline. Animals given DBS at 500 µA had 5.1 times more seizures than controls and a 2.8 fold increase in seizure rate as compared to preoperative values. In non-stimulated controls, the average frequency of seizures before and after surgery remained unaltered. *In vitro* recordings have shown that slices from animals previously given DBS at 100 µA had a longer latency for the development of epileptiform activity, shorter and smaller DC shifts, and a smaller spike amplitude compared to non-stimulated controls. In contrast, a higher spike amplitude was recorded in slices from animals given AN DBS at 500 µA.

## Introduction

Approximately 30% of patients with epilepsy continue to have seizures despite adequate medical treatment [1]. In this refractory population, surgery often comprises an effective therapeutic modality. Though resective procedures are still considered to be the surgical treatment of choice, some patients are not deemed to be good candidates because of multiple seizure foci, foci in eloquent brain regions or foci that cannot be identified. Under these circumstances, neuromodulation strategies represent a potential alternative [2]–[4].

Deep brain stimulation (DBS) involves the delivery of current to the brain parenchyma though implanted electrodes. Over the last decade, a series of preclinical and clinical studies have shown that DBS in the anterior nucleus of the thalamus (AN) reduces seizure rate and increases the latency for the development of seizures and status epilepticus (SE) [5]–[18]. To date, preclinical research in the field has largely been conducted in naïve rodents with seizures induced through the administration of chemical or electrical stimuli [6], [8], [13], [14], [18]–[20]. In the only study addressing the effects of DBS in chronic epileptic animals, AN stimulation had a proconvulsant effect [21].

We show that stimulation at parameters that approximate those used in clinical practice [22], [23] decreases the frequency of seizures and is associated with a reduction in hippocampal excitability. In contrast, DBS at high currents seems to be proconvulsant.

## Materials and Methods

Protocols were approved by the Animal Care committee of the Universidade Federal de São Paulo (2070/09).

### Surgery and AN stimulation

**Figure 1:**
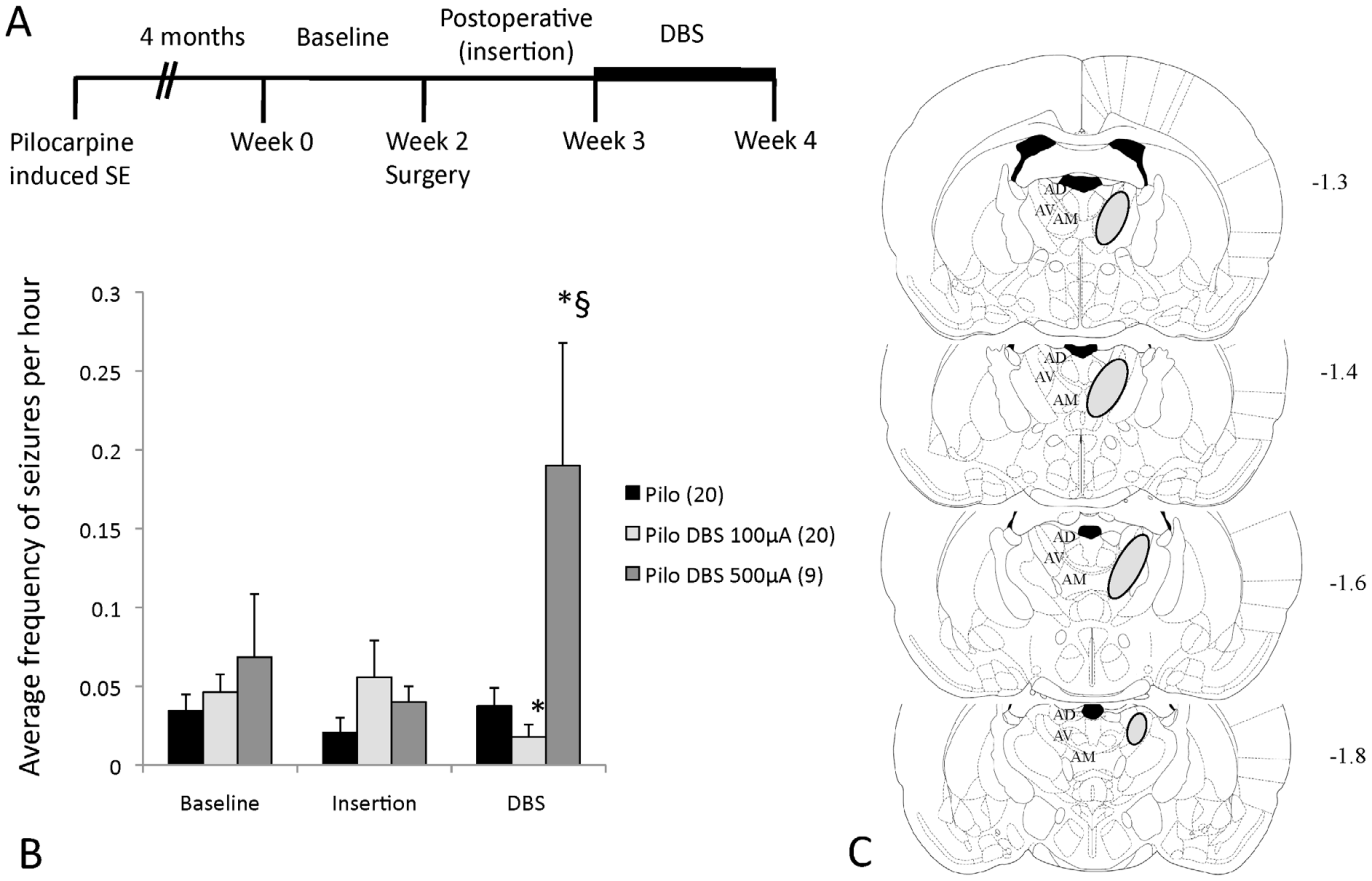
Effects of DBS on the frequency of spontaneous recurrent seizures in chronic epileptic rats. (A) Four months after pilocarpine-induced (Pilo) status epilepticus (SE), animals were videotaped for two weeks, followed by the implantation of anterior thalamic nucleus (AN) electrodes or sham surgery. On the first postoperative week, the frequency of behavioral seizures was recorded to study the effects of surgery and electrode insertion. On the second postoperative week, animals were given DBS (horizontal bar). (B) Animals treated with 100 µA had a 52% reduction in seizure rate as compared to sham-treated controls (p = 0.1) and 61% less seizures than at baseline (p = 0.05). In contrast, rats given DBS at 500 µA had 5.1 times more seizures than controls (p<0.01) and a 2.8 fold increase in seizure rate as compared to preoperative values (p = 0.03). (C) Schematic representation of coronal brain sections depicting the region in which the tips of the electrodes were identified. For clarity, we did not plot the tip of each of the 58 electrodes implanted in animals receiving stimulation but rather indicate the boundaries of the regions in which they were identified (circles). Values in B are presented as mean and SE. Numbers in parenthesis represent animals per group. In C, numbers on the right denote distance from the bregma. AD- anterodorsal nucleus of the thalamus; AV- anteroventral nucleus of the thalamus; AM- anteromedial nucleus of the thalamus. * statistically significant compared to preoperative values. § statistically significant compared to controls. Figure C was modified and reprinted from Paxinos and Watson, Copyright (1998) with permission from Elsevier.

Adult male Wistar rats (250–300 g) were injected with pilocarpine (Pilo; 320 mg/Kg i.p.). Four months after Pilo-induced SE, chronic epileptic rats were videotaped for 6 h/day, 5 days/week for 2 weeks to register their baseline frequency of behavioral seizures (Figure 1A). Animals were then paired according to seizure rate and assigned to receive either DBS or sham-surgery (holes drilled to the skull without the insertion of electrodes). Under ketamine/xylazine anesthesia (100/7.5 mg/kg i.p.), animals in the DBS group had insulated stainless steel electrodes (cathodes; 250 µm diameter; 0.5 mm exposed length) bilaterally implanted in the AN (anteroposterior −1.5, lateral±1.5, depth 5.2) [24]. A screw implanted over the right somatosensory cortex was used as the anode. On the first postoperative week, the effects of electrode insertion in the frequency of seizures were studied. On the second postoperative week, DBS was administered for 5 days (6 h/day) using a portable stimulator (St Jude Medical, Plano, TX) at 130 Hz, 90 µsec, and either 500 µA or 100 µA (Figure 1A). These settings were chosen as they were either effective against pilocarpine-induced seizures in our previous study (500 µA) [14] or estimated to generate a charge density (current x pulse width/area of exposed electrode) similar to that used in the clinic (100 µA) [5], [23], [25]. Stimulation frequency and pulse width were in the range of those used in clinical practice [5], [7], [9], [10].

A blinded investigator visually scored the frequency of behavioral seizures. Those characterized by clonic/tonic/tonic-clonic movements of the forelimbs culminating with rearing and falling were quantified from videotapes obtained during recording sessions.

### Electrophysiology

Prior to the experiments, different groups of chronic epileptic rats were given 5 days of DBS at the settings described above. Following CO_2_ narcosis, animals were decapitated. Their brains were removed from the skull and 400 µm hippocampal slices were cut on a vibratome. Slices were individually transferred to an interface-type chamber, placed on a membrane (0.4 µm Millicell culture plate inserts; Millipore, Badford, MA) and continuously bathed with artificial cerebrospinal fluid (aCSF; 127 mM NaCl, 2 mM KCl, 1.5 mM MgSO_4_, 1.1 mM KH_2_PO_4_, 26 mM NaHCO_3_, 2 mM CaCl_2_, and 10 mM glucose) at 33°C under a stream of moisturized 95% O_2_ – 5% CO_2_. One hour later, slices were perfused with a zero calcium and 8 mM potassium solution [26].

Extracellular field potentials were recorded from the hippocampal dentate gyrus (DG). Recording electrodes were made of microfilament capillary thin-walled glass (Clark Electromedical Instruments, GC150F-10) pulled with a DMZ-Universal Puller (Zeitz-Instruments, Germany). Electrodes were filled with 1 M NaCl (4–10 MΩ), connected to a head stage (model AI 402×50, ultralow *noise* amplifier – Axon Instruments, USA) and a biological amplifier (model Cyberamp 380 – Axon Instruments, USA). Recorded signals were filtered (3 kHz lowpass) and digitized (sampling frequency of 10 kHz) before the analysis.

For each group, slices containing the dorsal hippocampus of 5–9 animals were analyzed (2–3 slices per animal). After the development of epileptiform discharges, recordings were carried out for 20 minutes. The following parameters were considered for analysis: 1) Latency to record epileptiform discharges from the moment the bath was perfused with a zero calcium high potassium solution; 2) duration and amplitude of DC shifts; and 3) the amplitude of population spikes. A digital Fourier transform was used to quantify DC-shifts. Once in the frequency domain, the event signal was recalculated taking into account only components below 10 Hz. This process has allowed the analysis of DC shifts without the interference of population spikes. Event duration was calculated by subtracting the final time from the initial time of an event [26].

### Statistical Analysis and histology

ANOVA (Tukey post-hoc) was used to compare the frequency of seizures across groups. Comparisons between pairs of either animals given DBS at 100 µA and controls or the pre and postoperative frequency of seizures was carried out with a paired t-test. Mann Whitney U or Kruskall Wallis tests were used for the analysis of electrophysiology data. A Fisher exact test was used to compare proportions. Statistical significance was considered when p≤0.05. Only animals with electrodes implanted in the AN, as confirmed with cresyl-violet staining, were included in this study (Figure 1C). Our initial plan was to have 25 controls, 25 rats in the 100 µA DBS group and 10 animals in the group given DBS at 500 µA. We have missed the target in 3 animals receiving 100 µA (one rat had an electrode placed in the AN and the other in the ventral anterior nucleus, one rat had electrodes bilaterally implanted in the medial dorsal nucleus, and one rat had electrodes bilaterally placed in the dentate gyrus). These animals were excluded from the analysis along with their corresponding controls. One rat from the DBS 500 µA group has also been excluded due to misplaced electrodes (one electrode placed in the AN and the other in the ventral anterior nucleus). Aside from misplaced electrodes, two animals have died in the course of the experiments (one control and one rat from the DBS 100 µA group). Their mates were also excluded from the study. Due to all these losses, we ended our experiments with 20 animals in the groups receiving 100 µA DBS and sham treatment and 9 in the group treated with 500 µA.

## Results

### Effects of AN DBS *in vivo*

Our first experiment consisted in characterizing the effects of AN DBS against spontaneous recurrent seizures *in vivo*. Overall, we found that stimulation at 100 µA reduced seizure rate whereas 500 µA was proconvulsant (p<0.01). The proportion of animals that had a change in the frequency of seizures after surgery was also different across groups (p<0.01). In non-stimulated epileptic controls (n = 20), the average frequency of seizures before and after surgery remained unchanged (Figure 1B). In addition, when each animal was considered individually, we found that a similar proportion of rats had an increase (n = 7; 35%), a decrease (n = 7; 35%), or no change in the frequency of seizures before and after surgery (n = 6; 30%; Table S1).

Rats treated with AN DBS at 100 µA (n = 20) had a 52% reduction in the frequency of seizures as compared to sham-treated controls (p = 0.1) and 61% less seizures than at baseline (p = 0.05) (Figure 1B). Of the 20 animals treated, 12 (60%) had a decrease in seizure rate, 5 (15%) had the same number of seizures before and after DBS and 3 (10%) had an increase in the frequency of seizures while receiving stimulation (Table S1).

Animals given DBS at 500 µA (n = 9) had 5.1 times more seizures than controls (p<0.01) and a 2.8 fold increase in seizure rate as compared to preoperative values (p = 0.03) (Figure 1B). In this group, 8 of the animals treated with DBS (89%) had an increase in the number of seizures while receiving stimulation. In one animal (11%), the frequency of seizures in the postoperative period remained unchanged (Table S1).

### Effects of AN DBS *in vitro*

**Figure 2:**
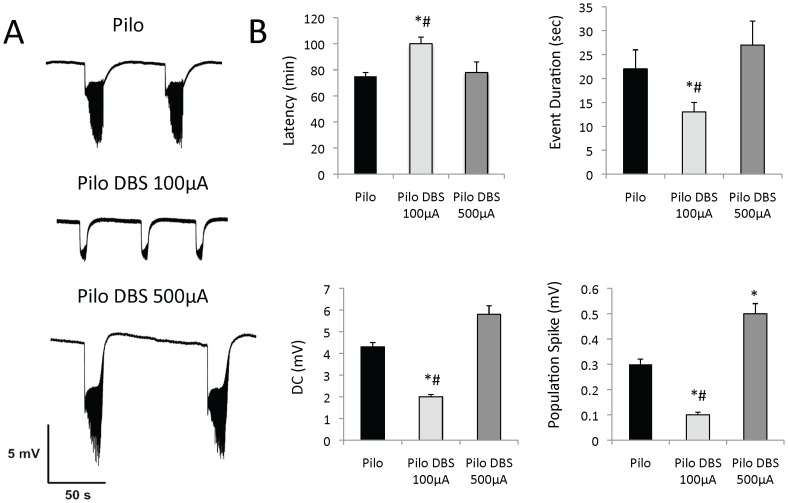
Effects of anterior thalamic nucleus (AN) stimulation *in vitro*. (A) Dentate gyrus extracellular activity in slices from chronic epileptic rats previously given sham surgery (n = 7), AN DBS at 100 µA (n = 9) or 500 µA (n = 5). When slices from different groups were perfused with a zero calcium high potassium solution, DC shifts intermingled with spiking discharges were promptly recorded. (B) Overall, slices from animals previously given DBS at 100 µA had a longer latency for the development of epileptiform activity, shorter and smaller DC shifts, and a smaller spike amplitude as compared to those from animals previously given no stimulation or DBS at 500 µA. In contrast, a higher spike amplitude was detected in slices from rats that had AN stimulation at 500 µA. * Statistically significant (p≤0.05) as compared to controls. # Statistically significant (p≤0.05) as compared to animals in the other DBS group.

Our initial hypothesis to explain the antiepileptic effects of AN DBS was that of a stimulation-induced decrease in hippocampal excitability. This was based on two fold. First, the AN is an important relay of the limbic circuitry having both direct and indirect connections with the hippocampus [27], [28]. Second, AN high frequency stimulation in rodents reduces neuronal firing rate in the hippocampal dentate gyrus [25]. To test this hypothesis, we recorded DG extracellular activity in slices from epileptic rats that did or did not receive 5 days of treatment with AN DBS. Epileptiform events have not been registered at baseline in either group. When slices were exposed to a zero calcium high potassium solution, however, DC shifts intermingled with spike discharges were clearly discriminated (Figure 2A).

Slices from animals previously given DBS at 100 µA had a longer latency for the development of epileptiform activity (p = 0.02; p = 0.01), shorter (p = 0.01; p = 0.01) and smaller DC shifts (p = 0.02; p = 0.01), and a smaller spike amplitude (p = 0.04; p = 0.02) when compared to those of non-simulated controls and animals treated with 500 µA, respectively (Figure 2B). In contrast, slices from animals previously given AN DBS at 500 µA had a higher spike amplitude as compared to controls (p = 0.02)(Figure 2B).

## Discussion

Our results suggest that AN DBS at a frequency, pulse width and amplitude that approximate those used in the clinic decreases seizure rate and reduces hippocampal excitability in chronic epileptic rats.

In our study, 60% of the animals given stimulation at 100 µA had a reduction in the number of seizures after receiving DBS. Further, seizure rate in this group was 61% and 52% lower than that recorded at baseline or in sham-treated controls. In contrast, animals given 500 µA had a 500% increase in seizure rate as compared to controls. Pilocarpine-induced seizures have been shown to progress over time in both intensity and frequency. To avoid a significant bias in this regard, we have studied epileptic rats four months following status, a timeframe during which the frequency of behavioral seizures in chronic epileptic animals tends to be more stable [29]. Another factor that deserves to be discussed is the choice of our control group. In the clinical scenario and in preclinical paradigms, behavioural changes following the implantation of electrodes (i.e. microlesion effect) are maximal immediately after surgery. In our study, animals implanted with DBS electrodes were given one week to recover from surgery before stimulation was commenced. During this time, the frequency of seizures was similar to that recorded in non-implanted controls. As we did not observe an effect of the insertion of the electrodes at this time point, we have decided not to pursue further experiments in sham treated rats with electrodes implanted.

In the only study published to date using AN DBS in chronic epileptic animals, Lado has studied the frequency of seizures in rats rendered epileptic following kainic acid injections [21]. He found that animals had an overall increase in the frequency of seizures while receiving stimulation. Differences between our study and his may be explained by the use of different animal models (pilocarpine vs. kainic acid) and stimulation parameters. Electrodes in our study had 250 µm in diameter, 0.5 mm of exposed surface, and an impedance of approximately 1.5–2 MΩ, while Lado has used constant voltage stimulation with 7 MΩ electrodes [21]. Though his output current amplitude was slightly lower than ours (140–550 µA) [21], with high impedance electrodes Lado was likely delivering either a higher charge density or influencing a smaller volume of tissue. In other words, he was likely delivering a higher current to a smaller volume of tissue. In line with this conclusion, we found that chronic epileptic rats given DBS at 500 µA had a significant increase in seizure rate.

An important aspect of our study was that 10% of the animals in the group given DBS at 100 µA had an increase in the frequency of seizures while receiving stimulation. The location of electrodes in these animals and the preoperative seizure rate were similar to those recorded in rats that had an improvement after DBS. At present, we have not been able to explain these findings. In a parallel scenario, a few patients reported in clinical trials had an increase in the frequency of seizures within the first days/weeks of treatment [5].

Our results expose an important aspect of chronic epileptic models, that is, the marked variability observed in the frequency of spontaneous recurrent seizures [29]. Even with a 52% difference in seizure rate between animals receiving 100 µA and non-stimulated controls, we were unable to find significant differences between groups. Seizure severity also seemed to be similar across groups, though we have only recorded tonic/clonic/tonic-clonic events that ultimately led to falls. In contrast to these findings, measures of variability in our electrophysiological experiments were not as pronounced. In fact, differences across groups could be easily detected and quantified. This suggests that better strategies to measure the effects of DBS *in vivo* are still needed. Alternatives could be the quantification of electrographic seizures and interictal spikes. Whether these will provide a more appropriate metric for studying the effects of DBS in rats remains to be demonstrated.

In addition to the reasons described in the methods section, a few additional aspects regarding stimulation settings need to be discussed in further detail. Though bipolar stimulation has been used in recent clinical trials [5], most applications of DBS use monopolar stimulation. Provided a similar electrode is used, both configurations generate different stimulation fields (one is more spherical and the other pear-shaped). In animal models, we have shown that the use of monopolar or bipolar stimulation did not influence the development pilocarpine-induced seizures or status epilepticus [14]. In this context, we find it unlikely that the use of bipolar stimulation in chronic epileptic rats might have significantly altered our findings. Another aspect is the use of constant current versus constant voltage. In theory, constant current (as used in our study) is much more reliable and theoretically more appropriate. Older stimulators used in most clinical trials are limited in this regard. In practical terms, the main disadvantage of constant voltage occurs in the first weeks/months of programming, while the impedance of the electrodes fluctuates. After this phase, both systems seem to be equivalent. Finally, in contrast to the clinical scenario in which patients receive constant DBS, animals in our study were stimulated for 6 h/day. Our current DBS system is external (i.e. animals are connected to stimulators through cables), which is sometimes problematic when animals develop seizures. In this sense, our experiments have always been conducted during the day, under the direct supervision of one of the investigators. It is possible that a more striking effect could have been observed if animals were continuously stimulated.

Previous reports have shown that electrical stimulation can abort seizures in animal models of epilepsy and humans [8], [15]. Rather than studying the direct consequences of stimulation *in vitro*, we decided to investigate functional changes associated with DBS given to chronic epileptic rats prior to electrophysiological experiments. Using this approach we expected to show that, in addition to the previously demonstrated disruption in seizure progression [8], [15], DBS could also induce a state of lower excitability and susceptibility for the development of ictal events. We decided to focus on the hippocampus rather than the AN as both structures are interconnected relays of the limbic circuitry [27], [28] and AN DBS has been shown to reduce DG firing rate in rodents [25]. When perfused with artificial CSF, epileptiform activity has not been recorded in slices from any of the tested groups. Significant differences, however, were noticed when slices from groups previously given DBS or sham treatment were exposed to a zero calcium high potassium solution. As in our behavioral experiments, we found that stimulation at 100 µA was protective, whereas 500 µA was proconvulstant. Though we did not explore mechanisms for the effects of DBS, the fact that the reduced excitability of DG cells was recorded when slices were exposed to a zero calcium solution suggest that non-synaptic mechanisms may play a role. Computer simulation studies have shown that in slices bathed with the solution used in our study, the transition from interictal to ictal states is dependent on the activity of Na+/K+ ATPase [26]. One of the potential mechanisms of DBS could then be to increase the activity of this enzyme, which would then lead to a reduction in hippocampal excitability. Though this has not been demonstrated so far, the administration of electrical stimulation to both thalamic and hippocampal slices from naïve animals does induce ATP and adenosine release [30]–[33]. These however, are only theoretical considerations that need to be proven in further experiments.

## Conclusions

In summary, we show that AN DBS at specific settings reduces the frequency of seizures and hippocampal excitability in chronic epileptic rodents. This may give us a basis to further investigate biological substrates of this therapy and understand how DBS works.

## Supporting Information

Table S1 **Frequency of seizures per hour for each individual animal before surgery and after receiving deep brain stimulation.**
(DOCX)

## References

[1] Sander JW (1993) Some aspects of prognosis in the epilepsies: a review. Epilepsia 34: 1007–1016. 8243349 10.1111/j.1528-1157.1993.tb02126.x

[2] Al-Otaibi FA, Hamani C, Lozano AM (2011) Neuromodulation in epilepsy. Neurosurgery 69: 957–979; discussion 979. 21716154 10.1227/NEU.0b013e31822b30cd

[3] Hamani C, Andrade D, Hodaie M, Wennberg R, Lozano A (2009) Deep brain stimulation for the treatment of epilepsy. Int J Neural Syst 19: 213–226. 19575509 10.1142/S0129065709001975

[4] Theodore WH, Fisher RS (2004) Brain stimulation for epilepsy. Lancet Neurol 3: 111–118. 14747003 10.1016/s1474-4422(03)00664-1

[5] Fisher R, Salanova V, Witt T, Worth R, Henry T, et al (2010) Electrical stimulation of the anterior nucleus of thalamus for treatment of refractory epilepsy. Epilepsia 51: 899–908. 20331461 10.1111/j.1528-1167.2010.02536.x

[6] Hamani C, Ewerton FI, Bonilha SM, Ballester G, Mello LE, et al (2004) Bilateral anterior thalamic nucleus lesions and high-frequency stimulation are protective against pilocarpine-induced seizures and status epilepticus. Neurosurgery 54: 191–195; discussion 195–197. 14683557 10.1227/01.neu.0000097552.31763.ae

[7] Hodaie M, Wennberg RA, Dostrovsky JO, Lozano AM (2002) Chronic anterior thalamus stimulation for intractable epilepsy. Epilepsia 43: 603–608. 12060019 10.1046/j.1528-1157.2002.26001.x

[8] Mirski MA, Rossell LA, Terry JB, Fisher RS (1997) Anticonvulsant effect of anterior thalamic high frequency electrical stimulation in the rat. Epilepsy Res 28: 89–100. 9267773 10.1016/s0920-1211(97)00034-x

[9] Andrade DM, Zumsteg D, Hamani C, Hodaie M, Sarkissian S, et al (2006) Long-term follow-up of patients with thalamic deep brain stimulation for epilepsy. Neurology 66: 1571–1573. 16540602 10.1212/01.wnl.0000206364.19772.39

[10] Kerrigan JF, Litt B, Fisher RS, Cranstoun S, French JA, et al (2004) Electrical stimulation of the anterior nucleus of the thalamus for the treatment of intractable epilepsy. Epilepsia 45: 346–354. 15030497 10.1111/j.0013-9580.2004.01304.x

[11] Lee KJ, Jang KS, Shon YM (2006) Chronic deep brain stimulation of subthalamic and anterior thalamic nuclei for controlling refractory partial epilepsy. Acta Neurochir (Suppl 99) 87–91. 17370771 10.1007/978-3-211-35205-2_17

[12] Lim SN, Lee ST, Tsai YT, Chen IA, Tu PH, et al (2007) Electrical stimulation of the anterior nucleus of the thalamus for intractable epilepsy: a long-term follow-up study. Epilepsia 48: 342–347. 17295629 10.1111/j.1528-1167.2006.00898.x

[13] Takebayashi S, Hashizume K, Tanaka T, Hodozuka A (2007) Anti-convulsant effect of electrical stimulation and lesioning of the anterior thalamic nucleus on kainic acid-induced focal limbic seizure in rats. Epilepsy Res 74: 163–170. 17448643 10.1016/j.eplepsyres.2007.03.007

[14] Hamani C, Hodaie M, Chiang J, del Campo M, Andrade DM, et al (2008) Deep brain stimulation of the anterior nucleus of the thalamus: effects of electrical stimulation on pilocarpine-induced seizures and status epilepticus. Epilepsy Res 78: 117–123. 18083005 10.1016/j.eplepsyres.2007.09.010

[15] Osorio I, Frei MG, Sunderam S, Giftakis J, Bhavaraju NC, et al (2005) Automated seizure abatement in humans using electrical stimulation. Ann Neurol 57: 258–268. 15668970 10.1002/ana.20377

[16] Cooper IS, Upton AR, Amin I, Garnett S, Brown GM, et al (1984) Evoked metabolic responses in the limbic-striate system produced by stimulation of anterior thalamic nucleus in man. Int J Neurol 18: 179–187. 6242978

[17] Upton AR, Cooper IS, Springman M, Amin I (1985) Suppression of seizures and psychosis of limbic system origin by chronic stimulation of anterior nucleus of the thalamus. Int J Neurol 19–20: 223–230. 2980675

[18] Zhang Q, Wu ZC, Yu JT, Zhong XL, Xing YY, et al (2012) Anticonvulsant effect of unilateral anterior thalamic high frequency electrical stimulation on amygdala-kindled seizures in rat. Brain Res Bull 87: 221–226. 22178354 10.1016/j.brainresbull.2011.11.023

[19] Takebayashi S, Hashizume K, Tanaka T, Hodozuka A (2007) The effect of electrical stimulation and lesioning of the anterior thalamic nucleus on kainic acid-induced focal cortical seizure status in rats. Epilepsia 48: 348–358. 17295630 10.1111/j.1528-1167.2006.00948.x

[20] Zhong XL, Lv KR, Zhang Q, Yu JT, Xing YY, et al (2011) Low-frequency stimulation of bilateral anterior nucleus of thalamus inhibits amygdale-kindled seizures in rats. Brain Res Bull 86: 422–427. 21893168 10.1016/j.brainresbull.2011.08.014

[21] Lado FA (2006) Chronic bilateral stimulation of the anterior thalamus of kainate-treated rats increases seizure frequency. Epilepsia 47: 27–32. 16417528 10.1111/j.1528-1167.2006.00366.x

[22] Hamani C, Nobrega JN, Lozano AM (2010) Deep brain stimulation in clinical practice and in animal models. Clin Pharmacol Ther 88: 559–562. 20720537 10.1038/clpt.2010.133

[23] Hamani C, Temel Y (2012) Deep brain stimulation for psychiatric disease: contributions and validity of animal models. Sci Transl Med 4: 142rv148. 10.1126/scitranslmed.3003722 22786683

[24] Paxinos G, Watson C (1988) The rat brain in stereotaxic coordinates: Academic Press. 10.1016/0165-0270(80)90021-7 6110810

[25] Hamani C, Dubiela FP, Soares JC, Shin D, Bittencourt S, et al (2010) Anterior thalamus deep brain stimulation at high current impairs memory in rats. Exp Neurol 225: 154–162. 20558163 10.1016/j.expneurol.2010.06.007

[26] de Almeida AC, Rodrigues AM, Scorza FA, Cavalheiro EA, Teixeira HZ, et al (2008) Mechanistic hypotheses for nonsynaptic epileptiform activity induction and its transition from the interictal to ictal state–computational simulation. Epilepsia 49: 1908–1924. 18513350 10.1111/j.1528-1167.2008.01686.x

[27] Shibata H (1992) Topographic organization of subcortical projections to the anterior thalamic nuclei in the rat. J Comp Neurol 323: 117–127. 1385491 10.1002/cne.903230110

[28] Shibata H (1993) Direct projections from the anterior thalamic nuclei to the retrohippocampal region in the rat. J Comp Neurol 337: 431–445. 7506716 10.1002/cne.903370307

[29] Arida RM, Scorza FA, Peres CA, Cavalheiro EA (1999) The course of untreated seizures in the pilocarpine model of epilepsy. Epilepsy Res 34: 99–107. 10210024 10.1016/s0920-1211(98)00092-8

[30] Cunha RA, Vizi ES, Ribeiro JA, Sebastiao AM (1996) Preferential release of ATP and its extracellular catabolism as a source of adenosine upon high- but not low-frequency stimulation of rat hippocampal slices. J Neurochem 67: 2180–2187. 8863529 10.1046/j.1471-4159.1996.67052180.x

[31] Tawfik VL, Chang SY, Hitti FL, Roberts DW, Leiter JC, et al (2010) Deep brain stimulation results in local glutamate and adenosine release: investigation into the role of astrocytes. Neurosurgery 67: 367–375. 20644423 10.1227/01.NEU.0000371988.73620.4C PMC2919357

[32] Mohammad-Zadeh M, Mirnajafi-Zadeh J, Fathollahi Y, Javan M, Jahanshahi A, et al (2009) The role of adenosine A(1) receptors in mediating the inhibitory effects of low frequency stimulation of perforant path on kindling acquisition in rats. Neuroscience 158: 1632–1643. 19041928 10.1016/j.neuroscience.2008.11.008

[33] Bekar L, Libionka W, Tian GF, Xu Q, Torres A, et al (2008) Adenosine is crucial for deep brain stimulation-mediated attenuation of tremor. Nat Med 14: 75–80. 18157140 10.1038/nm1693

