# Supplementary material for: Effects of Anterior Thalamic Nucleus Deep Brain Stimulation in Chronic Epileptic Rats
Source: PLoS One. 2014 Jun 3;9(6):e97618. doi: 10.1371/journal.pone.0097618 (PMC4043725; doi:10.1371/journal.pone.0097618)
Supplement: Table S1 — Frequency of seizures per hour for each individual animal before surgery and after receiving deep brain stimulation. (DOCX) [file pone.0097618.s001.docx]

**Table S1.** Frequency of seizures per hour for each individual animal before surgery and after receiving deep brain stimulation.

| **Pilo** | **Preoperative** | **Postoperative** |
| --- | --- | --- |
| rat 1 | 0.016203704 | 0.04 |
| rat 2 | 0.013888889 | 0 |
| rat 3 | 0.013888889 | 0.04 |
| rat 4 | 0.043981481 | 0.02 |
| rat 5 | 0.111111111 | 0.16 |
| rat 6 | 0.05 | 0.025 |
| rat 7 | 0 | 0 |
| rat 8 | 0.025 | 0.025 |
| rat 9 | 0 | 0 |
| rat 10 | 0.15 | 0.02 |
| rat 11 | 0 | 0 |
| rat 12 | 0 | 0 |
| rat 13 | 0 | 0.05 |
| rat 14 | 0 | 0.11 |
| rat 15 | 0.13 | 0.04 |
| rat 16 | 0.037037037 | 0 |
| rat 17 | 0 | 0 |
| rat 18 | 0 | 0.05 |
| rat 19 | 0.067 | 0.17 |
| rat 20 | 0.033 | 0 |
|  |  |  |
| **Pilo DBS 100µA** | **Pre-DBS** | **Post-DBS** |
| rat 1 | 0.006944444 | 0 |
| rat 2 | 0.006944444 | 0 |
| rat 3 | 0.013888889 | 0.095 |
| rat 4 | 0.020833333 | 0.0125 |
| rat 5 | 0.04 | 0.11 |
| rat 6 | 0.12 | 0.0125 |
| rat 7 | 0.1 | 0 |
| rat 8 | 0 | 0 |
| rat 9 | 0.1 | 0 |
| rat 10 | 0.025 | 0 |
| rat 11 | 0 | 0 |
| rat 12 | 0 | 0 |
| rat 13 | 0 | 0 |
| rat 14 | 0.1 | 0 |
| rat 15 | 0.05 | 0 |
| rat 16 | 0.08 | 0 |
| rat 17 | 0.13 | 0.07 |
| rat 18 | 0.133333333 | 0 |
| rat 19 | 0 | 0.06 |
| rat 20 | 0 | 0 |
|  |  |  |
| **Pilo DBS 500µA** | **Pre-DBS** | **Post-DBS** |
| rat 1 | 0.025 | 0.06 |
| rat 2 | 0 | 0.18 |
| rat 3 | 0.075 | 0.3 |
| rat 4 | 0 | 0 |
| rat 5 | 0.05 | 0.08 |
| rat 6 | 0.133333333 | 0.266666667 |
| rat 7 | 0.066666667 | 0.366666667 |
| rat 8 | 0 | 0.166666667 |
| rat 9 | 0.266666667 | 0.3 |
